# Latent class/profile analysis in research on school bullying behavior patterns: a systematic review

**DOI:** 10.3389/fpubh.2026.1837704

**Published:** 2026-09-23

**Authors:** Qingfan Qian, Chen Shen, Fangdu Liu

**Affiliations:** 1 Department of Science and Technology, Taizhou People's Hospital, Taizhou, Jiangsu, China; 2 Department of Nursing, Taizhou People's Hospital, Taizhou, Jiangsu, China; 3 Department of Public Health, Taizhou Hospital of Traditional Chinese Medicine, Taizhou, Jiangsu, China

**Keywords:** bullying behavior patterns, latent class analysis, latent profile analysis, school bullying, systematic review

## Abstract

**Background:**

School bullying is a major public health concern affecting adolescents worldwide. Traditional cutoff point and outlier approaches have inherent limitations in identifying bullying behavior patterns and fail to capture the heterogeneity of bullying roles and the co-occurrence of bullying types. Latent class/profile analysis (LCA/LPA), as a person-centered analytical approach, offers a novel pathway to deepen understanding of complex bullying behavior patterns.

**Methods:**

A systematic search of PubMed, Web of Science, CNKI, and Wanfang databases identified 1,071 records. Original empirical studies using LCA/LPA to investigate school bullying patterns were included. Information on study design, sample characteristics, model fit indices, class labels, and associated variables was extracted.

**Results:**

A total of 113 studies were included. LCA/LPA identified three core typological features in school bullying patterns: heterogeneous patterns of victims, heterogeneous patterns of bullies, and complex subtypes of bully-victims. Research has also extended to the co-occurrence of bullying with other behaviors (substance use, discrimination experiences, adverse childhood, etc.), as well as the differential associations of risk factors (gender, age, social support, family factors, etc.) and negative health outcomes with distinct bullying patterns. Bully-victims were further differentiated into multiple subtypes and exhibited the most severe internalizing and externalizing problems.

**Conclusion:**

LCA/LPA effectively reveals the heterogeneous patterns of school bullying and provides empirical evidence for the development of individualized intervention strategies. Future work should unify measurement tools, cover more bullying subtypes, and adopt longitudinal designs to track pattern changes over time.

## 1 Introduction

Bullying among students has been recognized as a major public health and social issue affecting school-aged adolescents worldwide (1). A survey conducted across 144 countries and regions revealed that one in three students (32%) had experienced bullying victimization, and among countries with trend data, nearly one-fifth reported a rising prevalence of bullying (2). In many Western countries, approximately 4–9% of school-aged children report frequent involvement in bullying perpetration, while 9–25% report being victimized (3). The problem has also become increasingly serious in Chinese schools. A study involving 15,415 secondary school students across 11 provinces and cities in China found that 10.89% reported being bullied, 1.38% reported bullying others, and 2.72% reported both victimization and perpetration (4).

Three primary roles are typically identified in school bullying: bullies, victims, and bully-victims (those who are both perpetrators and targets). Bullying has adverse consequences for all involved adolescents. Victims are prone to sleep difficulties, headaches, anxiety, depression, suicidal ideation, suicidal behaviors, and alcohol misuse, whereas bullies show elevated risks of suicidal ideation and behavior, smoking, alcohol use, weapon-carrying, and school maladjustment (5). Compared to pure bullies or pure victims, bully-victims experience the most severe internalizing and externalizing problems (6). Recent research has shifted toward a systems perspective that encompasses broader group participation, focusing on the differentiated roles and interactions of various participants in bullying episodes—including bystanders, reinforcers, protectors, and outsiders (7, 8).

Bullying encompasses traditional forms (physical, relational, verbal, sexual, and property-based) as well as cyberbullying. Traditional and cyberbullying often overlap, such that adolescents involved in traditional bullying are also likely to participate in cyberbullying (9). Given the heterogeneity of bullying roles and the complexity of bullying types, it remains unclear whether distinct patterns of involvement (e.g., non-involvement, high involvement, or subgroups characterized by specific types of bullying) emerge when both roles and types are considered simultaneously. Research indicates that adolescents experiencing multiple types of victimization face heightened risks and poorer physical and mental health outcomes (10). Thus, it is essential to examine the heterogeneity of bullying roles and the co-occurrence of bullying types to inform the development of tailored intervention strategies.

Currently, most studies classify school bullying involvement using cutoff scores or extreme values to distinguish bullies, victims, and bully-victims. Although these methods provide insight into the frequency or degree of bullying and victimization, they lack standardized cutoffs and fail to capture the heterogeneity of bullying roles or the co-occurrence of different types. Therefore, alternative approaches that simultaneously account for both bullying roles and types are needed to gain a deeper understanding of the complexity and heterogeneity of bullying behaviors. Latent class/profile analysis (LCA/LPA) is a person-centered methodological approach for understanding bullying behaviors. It identifies latent classes based on participants’ observable response patterns. This approach does not rely on predefined cutoffs but instead assumes an underlying latent variable that determines class membership, allowing for the simultaneous consideration of both roles and types (11). LCA/LPA thus provides a more empirically grounded basis for classifying school bullying patterns and has gained increasing attention in research on health-related behaviors in recent years.

This study employs a systematic review approach to synthesize the application of LCA/LPA in research on school bullying behavior patterns, aiming to provide empirical evidence for the development of individualized bullying prevention and intervention strategies. The specific objectives are to: (1) synthesize the typological characteristics of bullying behavior patterns identified by LCA/LPA; (2) summarize patterns of co-occurrence between bullying and other behaviors; (3) identify risk factors and health outcomes associated with distinct bullying patterns; and (4) assess the quality of existing studies and identify directions for future research. Notably, because the bulk of LCA/LPA applications in this field employ cross-sectional designs—with only a small subset using longitudinal or repeated-measures approaches—methodological quality was appraised using a cross-sectional instrument; the applicability of this tool to the longitudinal subset is addressed in the Methods (Section 2.4) and Discussion (Section 4.4).

## 2 Methods

### 2.1 Search strategy

A systematic search was conducted in four electronic databases: PubMed, Web of Science, CNKI (China National Knowledge Infrastructure), and Wanfang Data Knowledge Service Platform. The search period covered from database inception to June 2026. To ensure reproducibility, the search strategy was adapted to the native syntax and field tags of each database: in PubMed, the Text Word (tw) field was searched, which covers titles, abstracts, and indexed terms; in Web of Science, the Topic (TS=) field was searched; and in CNKI and Wanfang Data, the “主题” (subject) field was searched. The complete database-specific search strings, including all Boolean operators and field tags, are provided in Supplementary material 1.

### 2.2 Inclusion and exclusion criteria

Studies were included if they met the following criteria: (1) original empirical research; (2) used LCA/LPA as the primary analytical method; (3) focused on classifying patterns of school bullying behaviors; and (4) involved school-enrolled students as participants.

Studies were excluded if they were: (1) conference abstracts, dissertations, reviews, or commentaries; (2) used only traditional classification methods (cutoff points or extreme values); (3) applied LCA/LPA solely for scale validation rather than behavior pattern identification; (4) duplicate publications or studies with overlapping data; or (5) not available in full text.

### 2.3 Study selection and data extraction

Two reviewers independently conducted the study selection process. Duplicate records were first removed, followed by an initial screening based on titles and abstracts, and a full-text review for final inclusion. Disagreements were resolved through discussion or consultation with a third reviewer. Data were extracted by one reviewer using a pre-designed standardized form, including: (1) basic study information (first author, publication year, country/region); (2) sample characteristics (sample size, age/grade, gender distribution); (3) bullying measurement tools and types of bullying assessed; (4) LCA/LPA model results (optimal number of classes, class labels, and conditional probability profiles); and (5) covariates/outcome variables included and their associated findings. All extracted data were subsequently verified for accuracy by a second reviewer; disagreements were resolved through discussion or consultation with a third reviewer.

### 2.4 Quality assessment

Methodological quality of the included studies was assessed using the JBI Critical Appraisal Checklist for Analytical Cross-Sectional Studies, developed by the Joanna Briggs Institute. Although the JBI checklist was originally designed for cross-sectional studies, it was applied to all included studies given that the majority adopted cross-sectional designs. This checklist comprises eight items that evaluate: the clarity of inclusion criteria, the adequacy of participant and setting descriptions, the reliability and validity of exposure and outcome measurements, the identification and control of confounding factors, and the appropriateness of statistical methods. Each item is judged as “Yes” (counting 1 point), “No,” “Unclear” or “Not Applicable” (all counting 0 points), and the total score ranges from 0 to 8 points. Based on the percentage of the score, the quality of the literature is classified into three levels: ≥70% (≥6 points) is considered high quality, 50–69% (4–5 points) is of medium quality, and <50% (≤3 points) is of low quality. Quality ratings were independently assigned by two reviewers, with discrepancies resolved through discussion or consultation with a third party. Of the 115 studies appraised, 113 (98.3%) were rated as high quality (JBI score ≥6/8; scores of 8/8 in 111 studies, 7/8 in one, and 6/8 in one), and two (1.7%) were rated as low quality (1/8 and 2/8); no studies were rated as medium quality. (Supplementary material 2).

## 3 Results

### 3.1 Study selection and basic characteristics

The initial search yielded 1,071 records. After removing duplicates, conference abstracts, and dissertations, 599 records remained. Following title and abstract screening, 244 records were excluded. Full-text review of the remaining 355 articles led to the exclusion of 223 records. An additional 17 articles were excluded due to unavailability of full text or overlapping data. Two studies with low quality (with scores of 1/8 and 2/8 respectively) were excluded. Eventually, a total of 113 studies met the inclusion criteria. The study selection process followed the PRISMA guidelines (Figure 1).

**Figure 1:**
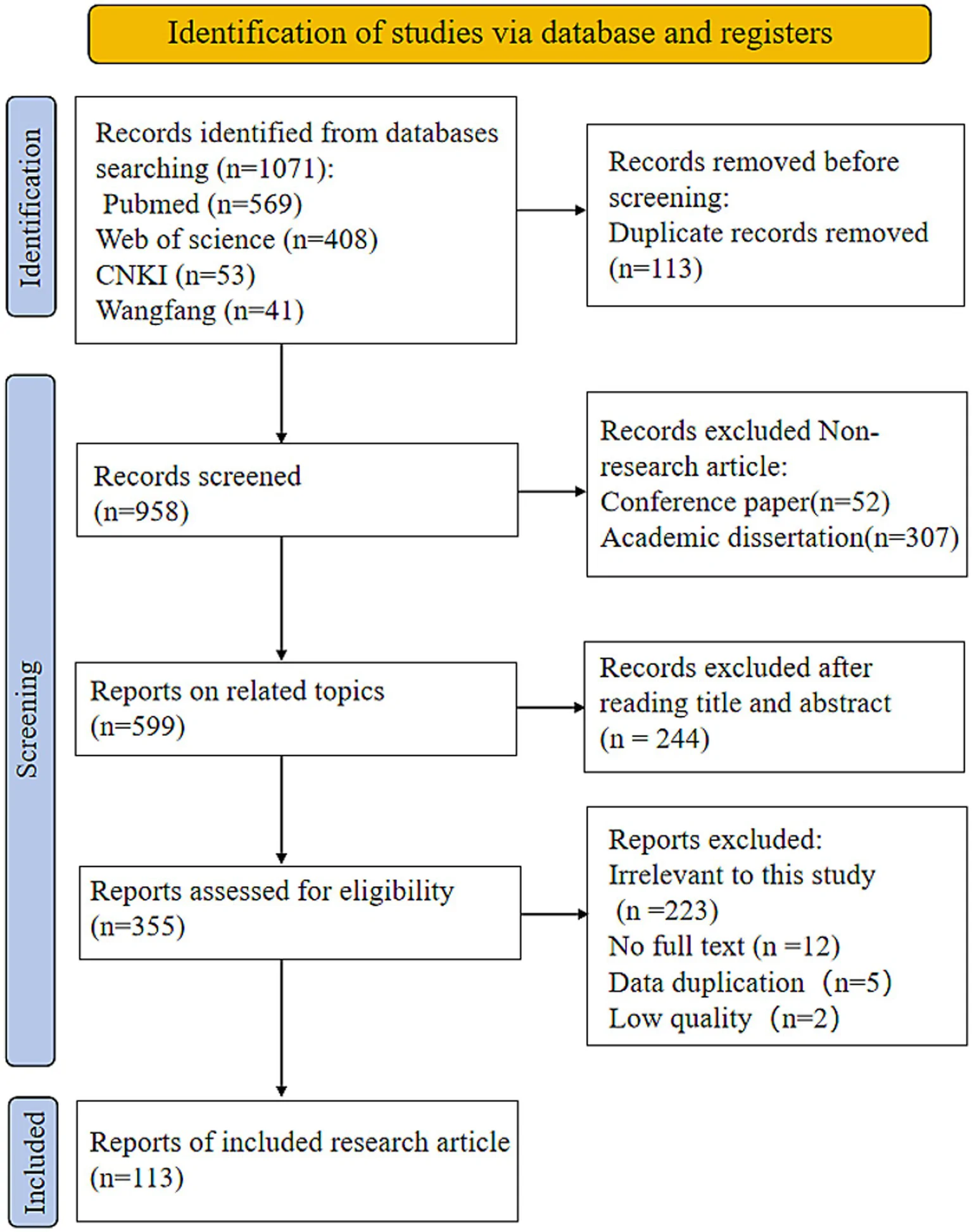
PRISMA flowchart.

The 113 included studies were published between 2008 and 2026, comprising 100 English-language and 13 Chinese-language articles. The studies covered a range of countries/regions: the United States (*n* = 48), China (*n* = 27), Spain (*n* = 8), and South Korea (*n* = 6) were the most common settings, followed by the Netherlands, Germany, and Slovenia (*n* = 2 each) and 14 other countries with one study each (Bangladesh, Belgium, Brazil, Canada, Finland, Hungary, Iran, Ireland, Italy, Morocco, Oman, Saudi Arabia, Singapore, and Turkey). The remaining four studies were multi-country investigations covering two to six countries each. Sample sizes ranged from 239 to 418,483 participants (median = 2,482; interquartile range = 997–6,562). The study populations spanned from kindergarten through university, with the majority focusing on middle and high school students. Table 1 summarizes the characteristics of the 113 included studies; study-level details are provided in Supplementary material 3.

**Table 1:** Characteristics of the 113 included studies.

Characteristic	Category	*n* (%)
Country	United States	48 (42.5)
	China	27 (23.9)
	Spain	8 (7.1)
	South Korea	6 (5.3)
	Germany	2 (1.8)
	Netherlands	2 (1.8)
	Slovenia	2 (1.8)
	Other countries	14 (12.4)
	Multi-country	4 (3.5)
Sample size	Range	239–418,483
	<1,000	28 (24.8)
	1,000–4,999	52 (46.0)
	5,000–9,999	13 (11.5)
	10,000–49,999	17 (15.0)
	≥50,000	3 (2.7)
Age group (school level)	Primary school or below	24 (21.2)
	Middle school	37 (32.7)
	High school	6 (5.3)
	University	9 (8.0)
	Primary and middle school	7 (6.2)
	Middle and high school	14 (12.4)
	Mixed (≥3 levels)	14 (12.4)
	Not applicable/not reported	2 (1.8)
Number of classes	2	2 (1.8)
	3	40 (35.4)
	4	49 (43.4)
	5	17 (15.0)
	6	3 (2.7)
	7	2 (1.8)
Role focus	Victimization only	12 (10.6)
	Perpetration only	14 (12.4)
	Both victimization and perpetration	73 (64.6)
	Bystander/defender	10 (8.8)
	Not classifiable	4 (3.5)

### 3.2 LCA/LPA model fit and determination of number of classes

All included studies commonly used the following fit indices to determine the optimal number of classes: the Akaike Information Criterion (AIC), Bayesian Information Criterion (BIC), sample-size adjusted BIC (aBIC), Entropy, Lo–Mendell–Rubin likelihood ratio test (LMR-LRT), and Bootstrap likelihood ratio test (BLRT). The final number of classes was determined jointly by model fit statistics and theoretical interpretability. Across the included studies, the number of retained classes ranged from 2 to 7: two-class solutions were retained in 2 studies (1.8%), three-class solutions in 40 studies (35.4%), four-class solutions in 49 studies (43.4%), five-class solutions in 17 studies (15.0%), six-class solutions in 3 studies (2.7%), and seven-class solutions in 2 studies (1.8%).

Notably, the fit indices reported by the included studies were heterogeneous: although most studies used information criteria (AIC, BIC, and/or aBIC) and entropy in combination with likelihood ratio tests (LMR-LRT and/or BLRT) to select the final model, the specific sets of indices and the values reported varied considerably across studies, and a number of studies did not report detailed fit-statistic values. This heterogeneity precluded a meaningful quantitative aggregation of fit-index values (e.g., typical BIC or entropy levels) across the included studies.

### 3.3 Typological characteristics of bullying behavior patterns

#### 3.3.1 Heterogeneous patterns of bullying victims

LCA/LPA studies consistently indicate that bullying victims are not a homogeneous group but can be differentiated into multiple subtypes based on the severity and types of victimization experienced (Table 2).

**Table 2:** Victimization subtypes identified in the included studies.

Study (author, year)	Country	Sample	No. of classes	Victimization subtypes
Ashrafi et al. (2020) (12)	Canada	*N* = 3,829 Age 9–14	3	Non-victimized; moderately victimized; aggressively victimized
Wang et al. (2010) (10)	USA	*N* = 7,475 (48.5% male) Grades 6–10	3	All-types victims; verbal/relational victims; non-victims
Berkowitz et al. (2015) (13)	California, USA	*N* = 418,483 (52.1% female) Grades 7, 9, 11	4	Frequent verbal, physical and sexual victimization; occasional verbal and physical victimization; verbal and sexual victimization; non-victimized
Lee et al. (2021) (14)	USA	*N* = 9,124 (51.7% male) Grades 5–10	3	Verbal-relational victims; all-type victims; noninvolved
Sideridis and Alghamdi (2023) (15)	Saudi Arabia	*N* = 3,867 (43.7% male) Grade 4	5	Low bullying; below average P-V-R; average bullying; above average P-V-R; high bullying
Al Saidi et al. (2025) (16)	Oman	*N* = 1,204 (604 girls, 50.2%) Aged 14–15	3	Nonvictims; traditional victims; dual victims
Malamut et al. (2021) (17)	Netherlands	*N* = 804 (50.2% male) Grades 7–9, Age *M* = 13.65 (range 11.29–16.75)	6	Convergent, low popularity; self-identified, high popularity; self-identified, average popularity; peer-identified, low popularity; non-victim, high popularity; non-victim, average popularity
Siu (2025) (18)	USA	*N* = 880 (45.8% male) Age *M* = 14.3 ± 1.85 (range 12–18)	5	Marginal victims; outpowered victims; classroom-specific victims; hallway-specific victims; pervasive victims
Zhang et al. (2014) (19)	China	*N* = 8,387 (54.0% male) Grades: 4th, 6th, and 8th	4	Verbal–physical victimization; Verbal–physical–relational victimization; Verbal–relational victimization; Non-victim
Xie et al. (2019) (20)	China	*N* = 3,761 (48.5% male) Age range = 11–20 years	4	All-types victimization; Traditional victimization; Mild traditional victimization; Non-victimization
Zhang et al. (2020) (21)	China	*N* = 20,722 (50.3% male) Mean age = 15.0 years	4/3	High victimization; Moderate victimization; Verbal victimization; Low victimization
Zhan et al. (2026) (22)	China	*N* = 414 (44.9% male) Mean age = 21.22 years	3	All-types victimization; verbal-only victimization; low victimization
Meehan et al. (2021) (23)	USA	*N* = 1,440 (50% male) Age *M* = 10.15 (range 8.17–12.50)	2/4	Non-victims; victims; moderate victims; discordant high victims; concordant high victims
Zhao et al. (2025) (25)	China	*N* = 1,667 (48.8% male) Mean age = 13.59 years	2	Low victimization; Traditional victimization
Yang et al. (2020) (26)	China	*N* = 2,015 at T1 (7th graders) *N* = 1,975 at T2 (8th graders)	4	Non-victimized; Low victimization; Moderate victimization; High victimization

Differentiation by severity: Nylund et al. (11) classified students’ victimization experiences into three patterns: high victimization, occasional victimization, and non-involvement. Ashrafi et al. (12) found similar patterns—high, moderate, and non-involvement—in a Canadian sample.

Differentiation by type: Wang et al. (10) examined the co-occurrence of five bullying types (physical, verbal, social exclusion, rumor spreading, and cyberbullying) and identified three classes: all-type victimization, verbal/relational victimization, and non-involvement. Berkowitz et al. (13) found four patterns among middle and high school students: frequent verbal/physical/sexual victimization; occasional verbal/physical victimization; verbal/sexual victimization; and non-victimization. Lee et al. (14) identified three patterns—verbal/relational, all-type, and non-involvement—across verbal, physical, relational, sexual, and cyber victimization. Sideridis and Alghamdi (15) found five patterns reflecting both severity differences and type overlap. Al Saidi et al. (16) identified three patterns among Omani students: poly-victimization (verbal, social, physical, and cyber), traditional victimization, and non-involvement. Beyond type and severity, Malamut et al. (17) distinguished victim subgroups by popularity—convergent victims with low popularity, self-identified victims with high popularity, and self-identified victims with average popularity. Siu (18) identified five victim subgroups based on power imbalance, bullying locations, and behavioral responses: marginal, outpowered, classroom-specific, hallway-specific, and pervasive victims. Several Chinese studies have also examined victimization patterns. Zhang et al. (19) categorized victims into four groups: verbal/physical, verbal/physical/relational, verbal/relational, and non-involved. Xie et al. (20), surveying 20 secondary schools across eight regions in Hunan Province, identified all-type, traditional, mild traditional, and non-victimization. Zhang et al. (21) found gender differences: females showed three classes (high, moderate, and low victimization), whereas males exhibited an additional verbal victimization pattern. Zhan et al. (22) examined cybervictimization among college students and identified all-type, verbal-only, and low cybervictimization.

Cross-temporal stability: Longitudinal studies have examined victimization patterns across educational stages. Meehan et al. (23) examined stability over one academic year, identifying two patterns at Time 1 (non-victims and victims) and four at Time 2 (non-victims, moderate victims, discordant high victims, and concordant high victims). Huang et al. (24) identified three trajectories—low-level victimization (consistently low), persistent victimization (consistently high), and moderate victimization (low in early years but high from middle school onward)—suggesting that middle school represents a peak period for bullying. Zhao et al. (25) found two patterns at both time points (low victimization and traditional victimization), with the low victimization group showing 95.7% stability and the traditional victimization group showing 40.9% stability. Yang et al. (26) identified four patterns at both time points: non-victimization, low, moderate, and high victimization.

#### 3.3.2 Heterogeneous patterns of bullies

Wang et al. (27) extended their previous research by examining the heterogeneity of bullies and found patterns consistent with their earlier victimization classification: all-type bullying, verbal/relational bullying, and non-involvement. Goldweber et al. (28) assessed bullying behavior patterns separately for middle and high school students and found slight differences between the two educational levels. A four-class model best fit the middle school data: low involvement, verbal bullying, high physical/verbal bullying, and high involvement. A three-class model best fit the high school data: low involvement, verbal bullying, and high involvement. These findings suggest that bullying patterns are more complex and diverse in lower grades, and that physical bullying behaviors decrease with age. Grunin et al. (29) specifically focused on cyberbullying to explore its complexity and identified four patterns: low, moderate, and high levels of cyberbullying, along with non-involvement. Kosir and Pivec (30) examined the “bistrategic control” mechanism whereby students simultaneously employ aggressive and prosocial behaviors, identifying five patterns: unpopular bullies, popular bullies, bistrategic, prosocial, and uninvolved. This revealed a complex interplay between bullying behavior and peer status—some bullies had high popularity while others were socially rejected. Sideridis and Alghamdi (15) investigated bullying patterns in elementary school students and identified five patterns, characterized by varying levels of low, medium, and high bullying experiences, with or without cyberbullying involvement, and differing levels of physical and verbal bullying (Table 3).

**Table 3:** Bullying perpetration subtypes identified in the included studies.

Study (author, year)	Country	Sample	No. of classes	Bullying perpetration subtypes
Wang et al. (2012) (27)	USA	*N* = 7,508 (48.5% male) Grades 6–10; Grades	3	All-types bullies; verbal/social bullies; non-involved
Goldweber et al. (2013) (28)	USA	*N* = 10,254 (50.2% male) Grades 6–8	4/3	Low involvement; verbal; high physical/high verbal; high involvement/low involvement; verbal; high involvement
Grunin et al. (2021) (29)	USA	*N* = 12,642 (51.45% male) Grades 5–10	4	No bullying; low cyberbullying element; moderate cyberbullying element; high cyberbullying element
Kosir and Pivec (2026) (30)	Slovenia	*N* = 6,379 (50% female) age *M* = 13.43 ± 0.64 (range 12–16)	5	Unpopular bullies; popular bullies; bistrategic; prosocial; uninvolved
Sideridis and Alghamdi (2023) (15)	Saudi Arabia	*N* = 3,867 (43.7% male) Grade 4	5	Low bullying; below average P-V-R; average bullying; above average P-V-R; high bullying

#### 3.3.3 Heterogeneous patterns of bully-victims

Given that bully-victims face the greatest negative health risks, differentiating subtypes within this population has become a research priority. Olivier et al. identified three characteristic patterns—uninvolved, victim-only, and victim-aggressor—across all grades and gender groups (31). Chung and Lee (32) conducted LCA on more than 3,000 eighth-grade students in South Korea and identified six subgroups: uninvolved, victim-only, bully-only, verbal bully-victim, social bully-victim, and all-type bully-victim. Coyle et al. (33) incorporated cyber, verbal, physical, and relational bullying and victimization and identified five patterns: uninvolved, traditional bully-victim, verbal bully-victim, traditional victim, and cyber bully-victim. Carmona-Rojas et al. (34), aiming to examine the overlap between traditional bullying and cyberbullying, simultaneously included both online and offline bullying/victimization behaviors and identified four patterns: traditional-cyber victims, traditional bully-victims, traditional-cyber bully-victims, and uninvolved, suggesting that although traditional bullying and cyberbullying co-occur, they remain relatively distinct behaviors. Aparisi et al. (35) identified three cyberbullying patterns among college students: not involved, victimized cyberbullies, and cyberbullies. Bettencourt et al. (36) explored how early bullying experiences affect long-term adaptation and identified three patterns: high involvement bully-victims, moderate involvement bully-victims, and low/no involvement youth. High bully-victims faced the greatest risks, including failure to complete high school on time and involvement in the criminal justice system. Several studies from China have contributed to this literature. Chen et al. (37), surveying middle school students in Suixi County, Anhui Province, identified three bully-victim classes: high bully-victim, high verbal/relational bully-victim, and low bully-victim. Ding et al. (38), surveying students across three cities in Anhui Province, identified four patterns: high bully-victim, moderate bully-victim, verbal/relational victim, and low involvement. Wu et al. (39) identified four patterns among rural elementary school students in Jiangxi Province: low involvement, moderate aggression-moderate victimization, low aggression-high victimization, and high aggression-high victimization. Lv et al. (40), in a large sample of students from Shenzhen, found three classes—uninvolved, victim-only, and bully-victim—and notably, no pure bully class emerged. Li et al. (41) found that males and females exhibited four and three patterns, respectively, and similarly found no pure bully class. Zhong et al. (42), studying students from 582 kindergartens, identified three patterns—not involved, physical bully-victim, and verbal-physical victim—extending the age range of research coverage and providing theoretical and empirical support for earlier intervention in bullying behaviors. Tian et al. (43) examined the developmental trajectories of cyberbullying perpetrators and victims from middle childhood to early adolescence and identified three patterns at all time points: non-involved, cyberbully-victims, and cybervictims (Table 4).

**Table 4:** Bully-victim subtypes identified in the included studies.

Study (author, year)	Country	Sample	No. of classes	Bully-victim subtypes
Olivier et al. (2022) (31)	Belgium	*N* = 3,903 Age *M* = 10.21–14.88	3	Uninvolved; victims; victim-aggressors
Chung and Lee (2020) (32)	South Korea	*N* = 3,478 (50.1% male) Grade 8	6	Uninvolved; victims; bullies; BV-V; BV-VS; BV-VSP
Coyle et al. (2021) (33)	USA	*N* = 799 (51.8% male) Grades 7–8	5	Not involved; traditional bully-victim; verbal bully-victim; traditional victim; cyber bully-victim
Carmona-Rojas et al. (2023) (34)	Spain	*N* = 2,639 (51.9% female) Mean age = 13.55 ± 1.28	4	Uninvolved; victims-cybervictims; victimized aggressors; wholly involved
Aparisi et al. (2023) (35)	Spain	*N* = 1,368 (36% male) Mean age = 21.34 ± 4.45	3	Not involved; victimized cyberbullies; cyberbullies
Bettencourt et al. (2023) (36)	USA	*N* = 594 (52.4% male) Grade 1 (*M* = 6.2 yrs)	3	High bully-victims; moderate bully-victims; no/low involvement
Chen et al. (2022) (37)	China	*N* = 1,687(60.4% male) Grade:7th	3	Low victimization–low perpetration; high verbal and relational victimization–perpetration; high victimization–high perpetration
Ding et al. (2023) (38)	China	*N* = 5,724 (52.5% male) Grades 7–9	4	Low involvement in bullying; verbal & relational victims; medium bully-victims; high bully-victims
Wu et al. (2023) (39)	China	*N* = 1,489 (53.7% male) Grades: 4th, 5th, 6th	4	Low involvement; moderate aggression–moderate victimization; low aggression–high victimization; high aggression–high victimization
Lv et al. (2022) (40)	China	*N* = 29,099 (52.1% male) Grades: 5th–6th, 7th–8th, 10th–11th	3	Non-involvement; victimization only; bully–victim
Li et al. (2023) (41)	China	*N* = 2,462 (53.9% male) Age range: 10–15 years	4/3	Low involvement; moderate involvement; victimization; high involvement
Zhong et al. (2022) (42)	China	*N* = 92,528 (53.8% male) Age range = 2 to 6 years	3	Not involved; physical bully-victim; verbal-physical victim
Tian et al. (2023) (43)	China	*N* = 4,326 (44.6% female) Age *M* = 9.94 ± 0.73 (range 8–13)	3	Non-involved; cybervictims; cyberbully-victims

#### 3.3.4 Heterogeneous patterns of bystanders and defenders

LCA/LPA has also been applied to examine the behavioral patterns of bystanders and defenders in bullying incidents. Yun identified four classes among Korean middle school students: assertive defenders, comforting defenders, sympathetic outsiders, and indifferent outsiders (7). Schultze-Krumbholz et al. (44) explored roles in cyberbullying and identified five classes among German students: prosocial defenders, communicating outsiders, aggressive defenders, bully-victims, and assistants. Tennant et al. (45) examined the overlap between defending behaviors and bullying involvement, identifying three classes: defenders, bully–victim–defenders, and students with low involvement in bullying, victimization, and defending. Kim et al. (46) identified four classes among fourth- to sixth-grade elementary school students: bullying passive bystanding, victimized active defending, uninvolved passive bystanding, and uninvolved active defending. Waasdorp and Bradshaw (47) identified five bystander patterns among high school students: passive, defender, contributor, limited, and inconsistent. Jia et al. (8) identified five cyberbullying bystander roles among Chinese college students: defensive bystanders, indifferent bystanders, low-involved bystanders, medium-involved bystanders, and high-involved bystanders (Table 5).

**Table 5:** Bystander/defender subtypes identified in the included studies.

Study (author, year)	Country	Sample	No. of classes	Bystander/defender subtypes
Yun (2020) (7)	South Korea	*N* = 1,373 (40% female) Age *M* = 14	4	Indifferent outsider; assertive defender; comforting defender; sympathetic outsider
Schultze-Krumbholz et al. (2018) (44)	Germany	*N* = 849 (52.7% female) Grades 7–10	5	Prosocial defenders; communicating outsiders; aggressive defenders; bully-victims; assistants
Tennant et al. (2019) (45)	USA	*N* = 700 (51% female) Grades 6–8	3	Low involvement; bully-victim-defenders; defenders
Kim et al. (2023) (46)	USA	*N* = 725 (50.6% male) Grades 4–6	4	Uninvolved active defending; uninvolved passive bystanding; bullying passive bystanding; victimized active defending
Waasdorp and Bradshaw (2018) (47)	USA	*N* = 18,863 (53.0% female) Age = 15.94 ± 1.3	5	Limited involvement; defender; passive; contributor; inconsistent
Jia et al. (2022) (8)	China	*N* = 1,025 (38.0% male) Age *M* = 20.05 ± 1.23 (range 17–23)	5	Defensive bystander; indifferent bystander; low-involved bystander; medium-involved bystander; high-involved bystander

### 3.4 Co-occurrence patterns of bullying with other behaviors

LCA/LPA enables the simultaneous inclusion of bullying-related variables and other behavioral variables in models, facilitating the exploration of co-occurrence patterns among multiple behaviors. Garnett et al. (48) examined the intersection of discrimination and bullying by simultaneously considering experiences of race-based, immigration-related, sexual orientation–based, and weight-based discrimination, as well as bullying victimization. Four distinct patterns were identified: low discrimination, racial discrimination, sexual orientation discrimination, and high-bullying racial and weight discrimination. This study demonstrated that discrimination and bullying co-occur among adolescents, and specific combinations of these experiences were associated with depressive symptoms, suicidal ideation, and deliberate self-harm. Luk et al. (49) investigated the co-occurrence of substance use (cigarettes, alcohol, marijuana) and bullying behaviors (physical, verbal, social exclusion, rumor spreading, cyberbullying) among U.S. adolescents and four patterns were identified: uninvolved, substance users, bullies, and substance-using bullies. Substance use and bullying co-occurred in 5.4% of adolescents, effectively identifying youth who may be engaged in multiple problem behaviors. Abbasi-Ghahramanloo et al. (50) identified five patterns among Iranian students aged 6–18 years: low risk, passive smoker, violence and aggression taker with passive smoking, violence and aggression taker without passive smoking, and high risk. Al-Mamun et al. (51) identified mental health risk profiles based on emotional symptoms, conduct problems, insomnia, suicidal behaviors, truancy, physical fighting, bullying, and peer/social isolation, finding three classes: low-risk, moderate-risk, and high-risk groups. El Kazdouh et al. (52) simultaneously incorporated violent behaviors, bullying, injury, suicidality, and substance use to identify health risk behaviors among Moroccan students, identifying four classes: low risk, violence dominant (high violence, low suicidality and substance use), moderate violence and high suicidality, and poly-risk (high violence, moderate suicidality, and high substance use). Several studies from China have also addressed co-occurrence patterns. Guo et al. (53) explored subgroups characterized by bullying victimization and daily interpersonal conflicts among middle school students, identifying three patterns: low victimization–low conflict, low victimization–high conflict, and high victimization–high conflict. The high victimization–high conflict group exhibited the highest levels of both non-lethal and lethal psychological crises. Liu et al. (54) examined co-occurrence patterns of adverse childhood experiences and victimization among middle school students in Anhui Province, identifying four classes: low-risk, family adversity, victimization, and high-risk groups. The group with co-occurring adverse experiences and bullying victimization reported the highest depressive symptom scores.

### 3.5 Predictors of bullying behavior patterns

LCA/LPA enables the incorporation of predictor variables into models via the AUXILIARY command, allowing for the identification of risk and protective factors associated with distinct bullying behavior patterns. This facilitates the development of targeted strategies to reduce risk factors and enhance protective factors. Coyle et al. (33) found that male students were more likely to be cyber bully-victims, whereas no significant gender differences were observed between traditional bully-victims and the uninvolved group. Higher academic achievement emerged as a protective factor against bullying involvement. Arens and Visser (55) reported that boys were more likely than girls to experience personal peer victimization, yet no significant gender differences were found in combined personal and ethnic peer victimization. Chung and Lee (32) found that among bully-victim subgroups, the proportion of females was higher in the Bully/Victim-Verbal and Social (BV-VS) group, while males outnumbered females in the Bully/Victim-Verbal (BV-V) and Bully/Victim-Verbal, Social, and Physical (BV-VSP) groups. The BV-V group demonstrated higher goal orientation, self-esteem, resilience, and self-control compared to the BV-VS and BV-VSP groups, and achieved the highest academic performance. Ferreira-Junior et al. (56) found that high bullying victimization and perpetration were significantly associated with alcohol use and poor academic performance; Black/Brown fifth-grade students were more likely to belong to the high bullying victimization class. Shao et al. (57), in a Chinese adolescent sample, found that boys were more likely to be bully-victims or bullies, and that peer support and teacher support served as protective factors. Ding et al. (58) reported that being male, younger age, higher depressive symptoms, lower classmate support, and greater affiliation with deviant peers increased the likelihood of belonging to the high traditional bully-victim class; being male, younger age, higher depressive symptoms, greater interparental conflict, and greater affiliation with deviant peers increased the likelihood of belonging to the high cyber bully-victim class.

### 3.6 Associations between bullying behavior patterns and health outcomes

LCA/LPA enables the incorporation of outcome variables into models via the AUXILIARY command, facilitating the examination of both short- and long-term negative consequences of bullying behaviors. This underscores the detrimental impact of bullying and highlights the importance of developing and implementing targeted intervention programs. Peras et al. (59) found that severe victims exhibited the poorest psychosocial outcomes, characterized by high levels of loneliness, negative affect, and poor social self-concept. Moderate victims resembled non-victims on most characteristics; however, their involvement in bullying perpetration suggested that they may represent a bully-victim subgroup. Bradshaw et al. (60) reported that students experiencing multiple forms of victimization exhibited more severe internalizing problems, externalizing problems, and retaliatory attitudes. Coyle et al. (33) found that students involved in bullying experienced more severe internalizing problems, externalizing problems, and school maladjustment, with bully-victims showing the most prominent emotional and behavioral difficulties. Arens and Visser (55) found that compared to non-victimized students, those who experienced peer victimization reported higher levels of anxiety and depression, as well as lower self-esteem and peer self-concept. Students who experienced personal peer victimization (without ethnic victimization) showed higher levels of school avoidance than those who experienced combined personal and ethnic peer victimization or no victimization. Kim et al. (46) found that students’ school connectedness and life satisfaction tended to decline when they experienced passive bystanding, victimization, or bullying. Moreover, the negative impact of victimization on life satisfaction may outweigh the protective effect of active defending behaviors. Méndez et al. (61) found that, compared to bullies or victims, bystanders demonstrated greater reading interest and more positive attitudes toward studying and collective reading.

## 4 Discussion

### 4.1 Advantages of LCA/LPA over traditional classification methods

This systematic review demonstrates that LCA/LPA offers significant advantages over traditional classification methods in research on school bullying behavior patterns. First, traditional cutoff/outlier approaches rely on researcher-defined thresholds [e.g., “two to three times per month” (62) or “one standard deviation above the mean” (63)] that lack standardization across studies, increasing the risk of misclassification. In contrast, LCA/LPA employs data-driven probabilistic models and objectively determines the optimal number of classes using fit indices, thereby circumventing biases introduced by subjective cutoffs. Second, traditional methods typically classify bullying roles along a single dimension (bully/victim/bully-victim), assuming homogeneity within each group. LCA/LPA, however, reveals substantial heterogeneity within each bullying role—victims can be further differentiated into high-, moderate-, or low-severity subtypes, as well as subtypes characterized by different combinations of bullying types. Similarly, bully-victims can be subdivided into multiple patterns distinguished by predominant bullying forms or victimization types. Third, LCA/LPA simultaneously incorporates both bullying roles and types, capturing the co-occurrence of different bullying forms, whereas traditional approaches tend to focus on overall bullying frequency while overlooking type-specific differences. The studies included in this review consistently indicate that the “all-type involvement” pattern and “specific-type involvement” patterns (e.g., verbal/relational only) differ in their predictors and health outcomes, representing a crucial complement to findings from traditional classification methods.

### 4.2 Core bullying behavior patterns identified by LCA/LPA and their implications

Synthesizing findings from 113 studies, the core behavior patterns identified by LCA/LPA in the field of school bullying can be summarized within the following typological framework:

Uninvolved group: This group emerged as the reference class across all studies, with proportions varying by research context (typically 40–85%). Adolescents in this group exhibited minimal or no involvement in bullying or victimization and demonstrated the most favorable mental health outcomes.

Victim-dominated patterns: These can be further subdivided into (1) high/all-type victimization group—characterized by exposure to multiple bullying types and reporting the highest levels of internalizing problems; (2) moderate/single-type victimization group—showing intermediate levels of risk; and (3) low-level victimization group—experiencing occasional mild victimization with relatively limited adverse effects.

Bully-dominated patterns: These patterns can be differentiated into high/all-type bullying groups, verbal/relational bullying groups, and specific-type bullying groups (e.g., cyberbullying), with externalizing problems being particularly prominent. Notably, multiple studies did not identify a pure bully class (34, 41), suggesting that bullying perpetration may rarely occur in isolation from victimization experiences.

Bully-victim mixed patterns: This represents the most complex category, which can be further divided into high-involvement subtypes (co-occurring all-type perpetration and victimization), traditional subtypes (involvement in traditional bullying and victimization only), traditional-cyber overlapping subtypes (co-occurring traditional and cyber forms), as well as victimization-dominant or perpetration-dominant subtypes. This group consistently exhibited the most severe internalizing and externalizing problems, making it a priority target for intervention.

Bystander/defender patterns: LCA/LPA also identified diverse bystander behavior patterns, including active defenders, passive outsiders, and bully assistants, providing empirical evidence for designing bystander-focused intervention strategies.

This typological framework deepens our understanding of bullying behaviors and provides an empirical foundation for tailored, pattern-specific interventions anchored in the correlates identified for each pattern. For the high/all-type victimization group, which consistently exhibited the poorest psychosocial outcomes—including loneliness, negative affect, and poor social self-concept (59)—interventions should prioritize trauma-focused psychological support; moreover, because students experiencing multiple forms of victimization also reported elevated retaliatory attitudes (60), this support should explicitly address anger and revenge motivations to prevent escalation into perpetration. For the high-involvement bully-victim group—found to show the most prominent emotional and behavioral difficulties of any class (33)—interventions need to address perpetration and victimization concurrently through emotion regulation and conflict resolution training, rather than treating the two roles separately. The repeated failure to identify a pure bully class across independent samples (34, 41) further implies that students referred for aggression management should also be screened for ongoing victimization experiences, given that perpetration rarely occurs in isolation from victimization. Finally, bystander-focused programs can be informed by the identified defender–outsider structure: because passive bystanding was itself associated with lower school connectedness and life satisfaction (46), mobilizing passive outsiders into defender roles may benefit not only victims but the bystanders themselves.

### 4.3 Extended applications of LCA/LPA in bullying research

This review also reveals that the application of LCA/LPA has expanded beyond mere classification of bullying behavior patterns into three important directions. First, LCA/LPA enables the investigation of co-occurrence patterns between bullying and other behaviors or experiences. It can identify shared patterns of bullying with substance use, discrimination experiences, and adverse childhood experiences, revealing the clustering of adolescent risk behaviors and suggesting that interventions should shift from single-behavior to multi-behavior comprehensive management. Second, LCA/LPA facilitates the analysis of predictors of bullying behavior patterns. By incorporating covariates, studies consistently identify significant associations between specific patterns and factors such as gender, age/grade, academic achievement, social support, family environment, and violence exposure, providing actionable targets for identifying high-risk populations and developing prevention strategies. Third, LCA/LPA enables the examination of associations between bullying behavior patterns and health outcomes. Findings indicate a graded distribution of health risks across patterns—from the uninvolved group, to single-pattern (victimization or perpetration) groups, to the bully-victim mixed pattern group—with mental health problems progressively worsening. This underscores the urgency of early identification of adolescents exhibiting mixed patterns of involvement.

### 4.4 Limitations and future directions

Although LCA/LPA has made significant progress in research on school bullying behavior patterns, several limitations remain that warrant attention in future studies. First, heterogeneity of measurement instruments. The included studies employed diverse bullying assessment questionnaires, varied reference timeframes (past month/past three months/past academic year), and differing definitions of bullying types, making direct comparisons of identified patterns challenging. Future research should promote the standardized application of validated bullying assessment tools in LCA/LPA studies and conduct cross-study integration and meta-analyses. Second, incomplete coverage of bullying types. Most studies focused on verbal, physical, relational, and cyberbullying, with notably insufficient attention to sexual and property-based bullying. This may result in the omission of unique behavior patterns among certain students. Subsequent research should incorporate a more comprehensive inventory of bullying types. Third, limitations in study design. The majority of existing studies employed cross-sectional designs, providing limited insight into the dynamic evolution of bullying behavior patterns. Although a few longitudinal studies suggest that middle school represents a critical window period, detailed characterization of pattern transition trajectories and turning points remains lacking. Future research should strengthen longitudinal tracking designs and incorporate Latent Transition Analysis (LTA) to examine individual movement patterns across different classes. Fourth, insufficient population coverage. Existing studies predominantly focus on middle and high school students, with relatively scarce LCA/LPA research on elementary school students (particularly lower grades) and college students. Given that the prevalence and manifestations of bullying behaviors may vary with age, extending the research scope to a broader age range is warranted. Fifth, lack of cross-cultural comparisons. Findings from different countries/regions suggest that cultural background may influence the presentation of bullying patterns; however, direct cross-cultural comparative studies are currently lacking. Future research should conduct multi-country collaborative LCA/LPA studies to explore the influence of cultural values, school climate, and anti-bullying norms on behavior patterns. Sixth, limitations of the review process itself. The absence of prospective protocol registration, the exclusion of grey literature, and the lack of systematic reference-list screening may limit the transparency of the review process and the completeness of study identification, despite the broad, bilingual database search. In addition, methodological quality was appraised with a single cross-sectional instrument (the JBI Analytical Cross-Sectional Checklist) regardless of study design. Although this ensured consistent and comparable ratings across the included literature, longitudinal-specific features (e.g., attrition, assessment intervals) were not captured, and the methodological quality of the small longitudinal subset may therefore be either over- or underestimated. Moreover, the included evidence was dominated by studies conducted in the United States (*n* = 48) and China (*n* = 27), which together account for 66.4% of the 113 included studies, and the search was restricted to English- and Chinese-language publications. The synthesized typologies may therefore reflect publication and language bias, and their generalizability to other cultural, linguistic, and educational contexts warrants caution.

## 5 Conclusion

This systematic review demonstrates that LCA/LPA, as a data-driven, person-centered analytical approach, effectively overcomes the limitations of traditional cutoff/outlier classification methods by simultaneously considering both bullying roles and types, thereby revealing the intrinsic heterogeneous structure of school bullying behaviors. Existing studies have identified core typological features including an uninvolved group, victim-dominated patterns (differentiated by severity and type), bully-dominated patterns (differentiated by severity and type), and bully-victim mixed patterns (the most complex and diverse), and have confirmed that distinct patterns are associated with differential risk factors and health outcomes. LCA/LPA has also been extended to broader domains such as bystander behavior patterns and the interplay between social status and bullying. The application of LCA/LPA in bullying research has expanded from behavioral pattern classification to co-occurrence behavior analysis, predictor identification, and outcome association testing, providing a scientific basis for developing stratified and tailored precision intervention strategies. Future research should focus on standardizing measurement instruments, incorporating more comprehensive bullying types, strengthening longitudinal designs to examine pattern evolution, expanding the scope of study populations, and conducting cross-cultural comparative studies, in order to further deepen the understanding of the complexity of school bullying behaviors and advance the refinement and precision of anti-bullying interventions.

## Data Availability

The original contributions presented in the study are included in the article/Supplementary material, further inquiries can be directed to the corresponding author.

## Supplementary material

SUPPLEMENTARY MATERIAL 1 Database-specific search strategy.

SUPPLEMENTARY MATERIAL 2 Quality assessment of included studies.

SUPPLEMENTARY MATERIAL 3 Characteristics of included studies.

## References

[1] Çalışkan Z Evgin D Bayat M Caner N Kaplan B Öztürk A. Peer bullying in the preadolescent stage: frequency and types of bullying and the affecting factors. J Pediatr Res. (2019) 6:169–79. doi: 10.4274/jpr.galenos.2018.26576

[2] United Nations Educational, Scientific and Cultural Organization. Behind the Numbers: Ending school Violence and Bullying. Paris, France: United Nations Educational, Scientific and Cultural Organization (2019).

[3] Menesini E Salmivalli C. Bullying in schools: the state of knowledge and effective interventions. Psychol Health Med. (2017) 22:240–53. doi: 10.1080/13548506.2017.1279740, 28114811

[4] Luo X Zheng R Xiao P Xie X Liu Q Zhu K . Relationship between school bullying and mental health status of adolescent students in China: a nationwide cross-sectional study. Asian J Psychiatr. (2022) 70:103043. doi: 10.1016/j.ajp.2022.103043 35219979

[5] Armitage R. Bullying in children: impact on child health. BMJ Paediatrics Open. (2021) 5:e000939. doi: 10.1136/bmjpo-2020-000939, 33782656 PMC7957129

[6] Liu J Guo SY Weissman R Liu HD. Investigating factors associated with bullying utilizing latent class analysis among adolescents. Sch Psychol Int. (2021) 42:11–32. doi: 10.1177/0143034320967061

[7] Yun HY. New approaches to defender and outsider roles in school bullying. Child Dev. (2020) 91:e814–32. doi: 10.1111/cdev.13312, 31580498

[8] Jia Y Wu Y Jin T Zhang L. How are bystanders involved in cyberbullying? A latent class analysis of the Cyberbystander and their characteristics in different intervention stages. Int J Environ Res Public Health. (2022) 19:16083. doi: 10.3390/ijerph192316083, 36498159 PMC9740610

[9] Wang CW Musumari PM Techasrivichien T Suguimoto SP Tateyama Y Chan CC . Overlap of traditional bullying and cyberbullying and correlates of bullying among Taiwanese adolescents: a cross-sectional study. BMC Public Health. (2019) 19:1756. doi: 10.1186/s12889-019-8116-z, 31888598 PMC6937625

[10] Wang J Iannotti RJ Luk JW Nansel TR. Co-occurrence of victimization from five subtypes of bullying: physical, verbal, social exclusion, spreading rumors, and cyber. J Pediatr Psychol. (2010) 35:1103–12. doi: 10.1093/jpepsy/jsq048, 20488883 PMC2980945

[11] Nylund K Bellmore A Nishina A Graham S. Subtypes, severity, and structural stability of peer victimization: what does latent class analysis say? Child Dev. (2007) 78:1706–22. doi: 10.1111/j.1467-8624.2007.01097.x, 17988316

[12] Ashrafi A Feng CX Neudorf C Alphonsus KB. Bullying victimization among preadolescents in a community-based sample in Canada: a latent class analysis. BMC Res Notes. (2020) 13:138. doi: 10.1186/s13104-020-04989-4, 32143693 PMC7060587

[13] Berkowitz R De Pedro KT Gilreath TD. A latent class analysis of victimization among middle and high school students in California. J Sch Violence. (2015) 14:316–33. doi: 10.1080/15388220.2014.980424

[14] Lee J Hong JS Tan K Pineros-Leano M Baek SA. Bullying victimization profiles of school-aged adolescents and associations with weight statuses: a latent class analysis. J Interpers Violence. (2021) 36:Np12949-np12972. doi: 10.1177/0886260520905087, 32046566

[15] Sideridis G Alghamdi MH. Profiling experiences of bullying in the elementary school: the role of gender. Children. (2023) 10:610. doi: 10.3390/children10040610, 37189859 PMC10137132

[16] Al Saidi A Longobardi C Fabris MA Mastrokoukou S Lin S. Exploring traditional and cyberbullying profiles in Omani adolescents: differences in internalizing/externalizing symptoms, prosocial Behaviors, and academic performance. Eur J Investig Health Psychol Educ. (2025) 15:100. doi: 10.3390/ejihpe15060100, 40558843 PMC12192131

[17] Malamut ST Dawes M van den Berg Y Lansu TAM Schwartz D Cillessen AHN. Adolescent victim types across the popularity status hierarchy: differences in internalizing symptoms. J Youth Adolesc. (2021) 50:2444–55. doi: 10.1007/s10964-021-01498-w, 34585323 PMC8580928

[18] Siu JCL. No safe space at school: exploring power dynamics, bullying locations, and maladaptive Behaviors among US adolescents. J Youth Adolesc. (2025) 54:2288–300. doi: 10.1007/s10964-025-02190-z, 40353997 PMC12420755

[19] Zhang X Li F Xiang Z Wang Y. Latent classes of campus victimization and anxiety among children and adolescents. Chin J Clin Psychol. (2014) 22:631–4. doi: 10.16128/j.cnki.1005-3611.2014.04.014

[20] Xie J Wei Y Zhu Z. Patterns of bullying victimization among adolescents in China: based on latent profile analysis. Psychol Dev Educ. (2019) 35:95–102. doi: 10.16187/j.cnki.issn1001-4918.2019.01.11

[21] Zhang S Gong M Li W Wang W Wu R Guo L . Patterns of bullying victimization and associations with mental health problems in Chinese adolescents: a latent class analysis. Int J Environ Res Public Health. (2020) 17:779. doi: 10.3390/ijerph17030779, 32012686 PMC7037478

[22] Zhan Q Zhang W Zhu C Evans R Zhang J Ma J. Cyberbullying in college: influencing factors and patterns of victimization. BMC Psychol. (2026) 14:994. doi: 10.1186/s40359-026-04580-9, 42129931 PMC13339837

[23] Meehan ZM Hubbard JA Grassetti SN Docimo MA Swift LE Bookhout MK. Using three reporters to identify pre-adolescent peer victims through latent profile analysis. Res Child Adolesc Psychopathol. (2021) 49:737–48. doi: 10.1007/s10802-021-00768-2, 33534095

[24] Huang X Zhou Y Yang R Li D Hu J Xue Y . Moderating role of mental health literacy on the relationship between bullying victimization during the life course and symptoms of anxiety and depression in Chinese college students. BMC Public Health. (2023) 23:1459. doi: 10.1186/s12889-023-16326-y, 37525159 PMC10388468

[25] Zhao Z Wu Z Liu G. Latent class transition of school bullying victimization among rural junior high school students in Guangxi. Zhongguo Xue Xiao Wei Sheng. (2025) 46:1534–8. doi: 10.16835/j.cnki.1000-9817.2025328

[26] Yang X Chen L Cao Y Bi X Chen G Zhang W. Peer victimization during early adolescence: person-centered analysis. Xin Li Fa Zhan Yu Jiao Yu. (2020) 36:146–56. doi: 10.16187/j.cnki.issn1001-4918.2020.02.03

[27] Wang J Iannotti RJ Luk JW. Patterns of adolescent bullying behaviors: physical, verbal, exclusion, rumor, and cyber. J Sch Psychol. (2012) 50:521–34. doi: 10.1016/j.jsp.2012.03.004, 22710019 PMC3379007

[28] Goldweber A Waasdorp TE Bradshaw CP. Examining the link between forms of bullying behaviors and perceptions of safety and belonging among secondary school students. J Sch Psychol. (2013) 51:469–85. doi: 10.1016/j.jsp.2013.04.004, 23870442 PMC6414047

[29] Grunin L Yu G Cohen SS. The relationship between youth cyberbullying Behaviors and their perceptions of parental emotional support. Int J Bullying Prevent. (2021) 3:227–39. doi: 10.1007/s42380-020-00080-5, 33005875 PMC7465883

[30] Košir K Pivec T. Prosocial, aggressive, or both? A multilevel latent profile analysis of peer status and social behavior in early adolescence. J Res Adolesc. (2026) 36:e70149. doi: 10.1111/jora.70149, 41588569 PMC12835455

[31] Olivier E Morin AJS Vitaro F Galand B. Challenging the "'mean kid"' perception: boys' and girls' profiles of peer victimization and aggression from 4th to 10th grades. J Interpers Violence. (2022) 37:Np15095-np15129. doi: 10.1177/0886260521997949, 33719703 PMC9465533

[32] Chung JY Lee S. Are bully-victims homogeneous? Latent class analysis on school bullying. Child Youth Serv Rev. (2020) 112:104922. doi: 10.1016/j.childyouth.2020.104922

[33] Coyle S Cipra A Rueger SY. Bullying types and roles in early adolescence: latent classes of perpetrators and victims. J Sch Psychol. (2021) 89:51–71. doi: 10.1016/j.jsp.2021.09.003, 34836576

[34] Carmona-Rojas M Ortega-Ruiz R Romera EM. Bullying and cyberbullying, what do they have in common and what not? A latent class analysis. An Psicol. (2023) 39:435–45. doi: 10.6018/analesps.516581

[35] Aparisi D Delgado B Bo RM. Latent profiles of cyberbullying among university students and its relationship with social anxiety and aggressiveness. Educ Inf Technol. (2023) 28:13269–85. doi: 10.1007/s10639-023-11725-5

[36] Bettencourt AF Clary LK Ialongo N Musci RJ. Long-term consequences of bullying involvement in first grade. J Sch Psychol. (2023) 97:63–76. doi: 10.1016/j.jsp.2023.01.004, 36914367 PMC10020929

[37] Chen S He Y Yuan M Li Y Chang J Wang G . Mediating role of depression symptom in the association among peer bullying and suicidal ideation in junior high school students. Chin J Sch Health. (2022) 43:1456–61. doi: 10.16835/j.cnki.1000-9817.2022.10.004

[38] Ding H Cao L Xu B Li Y Xie J Wang J . Involvement in bullying and sleep disorders in Chinese early adolescents. Front Psych. (2023) 14:1115561. doi: 10.3389/fpsyt.2023.1115561, 37181869 PMC10172573

[39] Wu M Song W Liang L. Types of aggressive victims of rural pupils and their school adaptation: based on latent profile analysis. Journal of East China Normal University. (2023) 41:40–9. doi: 10.16382/j.cnki.1000-5560.2023.01.004 (in Chinese)

[40] Lyu J Zheng H Fu L. Relationship between bullying victimization and perpetration, academic achievement and screen time among primary and secondary school students. Chin J Sch Health. (2022) 43:557–61. doi: 10.16835/j.cnki.1000-9817.2022.04.018 (in Chinese)

[41] Li X Liu Y Wang Y Jiang S. Effects of violence exposure on school bullying among early-age adolescents of different genders: based on latent profile analysis. Psychol Dev Educ. (2023) 39:255–65. doi: 10.16187/j.cnki.issn1001-4918.2023.02.12 (in Chinese)

[42] Zhong M Zhao HX Ai J Zeng ST Stone-MacDonald AK. Bullying and victimization in Chinese affordable kindergartens: a latent profile analysis. Early Child Educ J. (2022) 50:773–83. doi: 10.1007/s10643-021-01185-2

[43] Tian L Huang J Huebner ES. Profiles and transitions of cyberbullying perpetration and victimization from childhood to early adolescence: multi-contextual risk and protective factors. J Youth Adolesc. (2023) 52:434–48. doi: 10.1007/s10964-022-01633-1, 35648261

[44] Schultze-Krumbholz A Hess M Pfetsch J Scheithauer H. Who is involved in cyberbullying? Latent class analysis of cyberbullying roles and their associations with aggression, self-esteem, and empathy. Cyberpsychol J Psychosoc Res Cyberspace. (2018) 12:2. doi: 10.5817/CP2018-4-2

[45] Tennant JE Klossing JJ Demaray MK Dorio N Bixler T Jones C. Internalizing problems of youth involved in bullying via different participant role combinations and gender. Sch Psychol Rev. (2019) 48:222–36. doi: 10.17105/spr-2017-0078.v48-3

[46] Kim I Lee HY Hong JS Carney J Hazler RJ. Profiles of participation in school bullying: association with student well-being. J Couns Dev. (2023) 101:224–35. doi: 10.1002/jcad.12458

[47] Waasdorp TE Bradshaw CP. Examining variation in adolescent bystanders' responses to bullying. Sch Psychol Rev. (2018) 47:18–33. doi: 10.17105/spr-2017-0081.v47-1

[48] Garnett BR Masyn KE Austin SB Miller M Williams DR Viswanath K. The intersectionality of discrimination attributes and bullying among youth: an applied latent class analysis. J Youth Adolesc. (2014) 43:1225–39. doi: 10.1007/s10964-013-0073-8, 24318776

[49] Luk JW Wang J Simons-Morton BG. The co-occurrence of substance use and bullying behaviors among U.S. adolescents: understanding demographic characteristics and social influences. J Adolesc. (2012) 35:1351–60. doi: 10.1016/j.adolescence.2012.05.003, 22698675 PMC3432689

[50] Abbasi-Ghahramanloo A Heshmat R Safiri S Motlagh ME Ardalan G Mahdavi-Gorabi A . Risk-taking behaviors in Iranian children and adolescents: a latent class analysis approach: Caspian IV study. J Res Health Sci. (2018) 18:e00428. PMC6941635 30728314

[51] Al-Mamun F Mm AL Mamun MA. Identifying adolescent mental health and behavioral risk profiles: the role of childhood adversities, family environment, and peer support. Brain Behav. (2026) 16:e71531. doi: 10.1002/brb3.71531 PMC13247134 42260788

[52] El Kazdouh H Boubih S El-Ammari A El Achhab Y El-Ammari A Bouftini S. A latent class analysis of co-occurring substance use, suicidality, and violence-related behaviors among Moroccan adolescents. Int J Adolesc Med Health. (2026). doi: 10.1515/ijamh-2025-0154, 42204963

[53] Guo Y Li H Sun F Wei S. Patterns of interpersonal conflict and psychological crisis in adolescents: the mediating role of psychological pain. Journal of Psychological Science. (2025) 48:242–51. doi: 10.16719/j.cnki.1671-6981.20250123 (in Chinese)

[54] Liu Q Li Y Wang C Zhao Y Ren W Su P . Adverse childhood experiences, bullying victimization, and depressive symptoms among Chinese adolescents: the moderating role of perceived social support. BMC Public Health. (2026) 26:2194. doi: 10.1186/s12889-026-27896-y, 42192370 PMC13390311

[55] Arens AK Visser L. Personal peer victimization and ethnic peer victimization: findings on their co-occurrence, predictors, and outcomes from a latent profile analysis. Child Abuse Negl. (2020) 99:104250. doi: 10.1016/j.chiabu.2019.104250, 31835234

[56] Ferreira-Junior V Valente JY Sanchez ZM. Examining associations between race, gender, alcohol use, school performance, and patterns of bullying in the school context: a latent class analysis. J Interpers Violence. (2022) 37:Np12857-np12880. doi: 10.1177/0886260521999123, 33729046

[57] Shao A Liang L Yuan C Bian Y. A latent class analysis of bullies, victims and aggressive victims in Chinese adolescence: relations with social and school adjustments. PLoS One. (2014) 9:e95290. doi: 10.1371/journal.pone.0095290, 24740096 PMC3989267

[58] Ding Y Li DP Li X Xiao JL Zhang HY Wang YH. Profiles of adolescent traditional and cyber bullying and victimization: the role of demographic, individual, family, school, and peer factors. Comput Human Behav. (2020) 111:106439. doi: 10.1016/j.chb.2020.106439

[59] Peras I Pivec T Kalisnik A Kosir K. Identifying adolescents' victimization experiences: a latent profile analysis approach. Curr Psychol. (2025) 44:847–63. doi: 10.1007/s12144-024-07152-5

[60] Bradshaw CP Waasdorp TE Johnson SL. Overlapping verbal, relational, physical, and electronic forms of bullying in adolescence: influence of school context. J Clin Child Adolesc Psychol. (2015) 44:494–508. doi: 10.1080/15374416.2014.893516, 24738548

[61] Méndez I Gómez ER Ruiz-Esteban C Delgado MD García-Fernández JM, Profiles of bullying, cyberbullying, and disinterest in reading among primary school learners in Spain. Cyberpsychol J Psychosoc Res Cyberspace (2024) 18:10. doi: 10.5817/CP2024-4-10

[62] Gaffney H Ttofi MM Farrington DP. Effectiveness of school-based programs to reduce bullying perpetration and victimization: an updated systematic review and meta-analysis. Campbell Syst Rev. (2021) 17:e1143. doi: 10.1002/cl2.1143 PMC8356322 37131921

[63] Salmivalli C Nieminen E. Proactive and reactive aggression among school bullies, victims, and bully-victims. Aggress Behav. (2002) 28:30–44. doi: 10.1002/ab.90004

